# Supplementary material for: Participation in interventions and recommended follow-up for non-attendees in cervical cancer screening -taking the women’s own preferred test method into account—A Swedish randomised controlled trial
Source: PLoS One. 2020 Jul 2;15(7):e0235202. doi: 10.1371/journal.pone.0235202 (PMC7332065; doi:10.1371/journal.pone.0235202)
Supplement: S1 Data — (DOCX) [file pone.0235202.s002.docx]

**Forskningsplan**

**Riktade insatser till uteblivarna i cervixcancerscreening programmet**

**Bakgrund:**

Cervixcancer är den fjärde vanligaste cancern hos kvinnor i världen med 485 000 nya fall 2013 och orsaken till 236 000 dödsfall (1). Sedan introduktionen av cervixcancerscreening programmet på 1960-talet har incidensen av cervixcancer minskat betydligt i Sverige. Idag diagnostiseras ca 450 kvinnor/år med cervixcancer och under 40 år av cervixcancerscreening i Sverige har incidensen minskat med 67%(2).

Ett högt deltagande i cervixcancerscreening programmet är en avgörande framgångsfaktor för att förhindra uppkomsten av höggradiga cellförändringar och cervixcancer och ger ett närmare 90% skydd från att insjukna(3). Förutsättningen för att utveckla cellförändringar och cervixcancer är förekomsten av en kvarvarande humant papillomvirus (HPV) infektion under lång tid. Studier visar att ca 80% av de som dör i cervixcancer och ca 2/3 av de som insjuknar i cervixcancer uteblivit från rekommenderade kontroller. Förekomsten av höggradig dysplasi är ca 4 ggr så stor i den grupp som ej följer rekommenderade intervall jfr med de som följer screeningprogrammet ( 4).

Det finns olika anledningar till att kvinnor avstår från att komma för cellprovtagning efter kallelse skickats det handlar bla om oro, att man känner sig frisk, obekväm med situationen eller rädd för cancer. Tillgängligheten till mottagningen där provet utförs är också viktig. Deltagandet i cervixcancerscreeningsprogrammet är lägre bland ensamstående, lågutbildade och de som tillhör en lägre socioekonomisk grupp (5) . Nyinflyttade till Sverige deltar också i lägre omfattning (6).

Som **uteblivare** definieras i vissa studier de kvinnor som ej lämnat cellprov på mer än 6 år i åldern 30-49 år och mer än 8 år i åldern 50-65 år. Dessa utgör alltså en riskgrupp för att få cervixcancer och det är angeläget med riktade insatser för att öka hörsamheten till screeningprogrammet i denna grupp (4).

Processregistret i det nationella kvalitetesregistret för cervixcancerscreening den sk "cyt-burken", innehåller historiska data över varje kvinnas cellprover och behandlingar. Region Östergötland har under hösten 2015 anslutits till cytburken. Via denna funktion kan man söka ut och finna de som inte kommer på regelbunden cellprovstagning. Data från nationella kvalitetsregistret framtagna 2012 visar att uteblivargruppen är ca 12 000 kvinnor i Östergötland.

**Syftet med projektet är att :**

-öka deltagandet hos uteblivarna och på så sätt kunna erbjuda en höggrisk grupp för cellförändringar utredning och behandling

-undersöka vilken intervention som leder till högst deltagande i provtagning

- undersöka förekomsten av cellförändringar i de olika interventionsgrupperna ( se nedan)

-undersöka hur många av de som har en cellförändring också kommer på rekommenderad behandling/uppföljning

-undersöka orsaken till att de uteblivit från rekommenderade kontroller

**Metod:**

Vi avser att randomisera ca12000 kvinnor i Östergötland, definierade som "uteblivare"i cytburken till 3 olika grupper enligt följande:

1. HPV hemtest: Antal: 4000 st. Kvinnorna får ett informationsbrev samt ett kit för HPV hemtest hemskickat till sin folkbokföringsadress. Om HPV kittet ej är returnerat inom 1 månad skickas ett påminnelsebrev. Kvinnor som diagnostiseras med ett pos HPV test får ett svarsbrev hemskickat till sig samt en läkartid på KK mottagingen för cellprov + kolposkopi (undersökning av livmodertappen med hjälp av mikroskop). Kvinnor som diagnosticeras med ett neg HPV får ett svarsbrev hemskickat samt en uppmaning om att gå på sin nästa cellprovskontroll (kallas årligen i screeningprogrammet).
2. Telefonsamtal : Antal: 4000 st. Kvinnorna i denna grupp där telefonuppgifter från cosmic/obstetrix/internet kunnat identifieras får ett informationsbrev om att de kommer att bli uppringda av barnmorska inom 1 månad . Vid telefonsamtalet finns en guide för barnmorskan att utgå ifrån. Kvinnan kommer att bli erbjuden en tid för cellprov på Kvinnohälsan hos en barnmorska eller att få ett HPV hemtest skickat till sin folkbokföringsadress. De kvinnor som väljer HPV hemtest handläggs enligt punkt A. De kvinnor som väljer att komma för cellprov får en bokad tid och handläggs som en GCK patient. De kvinnor som avstår från båda interventionerna uppmanas komma och lämna cellprov vid nästa kallelse (kallas årligen i screeningprogrammet). Om telefonuppgifter ej kan hittas handläggs kvinnan enligt punkt C.
3. Väntelista: Antal: 4000st**. Denna grupp kommer att få en årlig påminnelse enligt rådande rutin på kvinnokliniken om att komma och ta cellprov. Denna årliga påminnelse skickas även ut till grupp A och B. Inget informationsbrev kommer utgå från projektet. Vi önskar dock kunna ta fram antal cellprov och resultaten av cellrpvoerna som tas i denna grupp inom 2 år från studiestart för att se om någon av interventionerna A eller B är mer effektiv än den redan rådande. Dvs göra en kvalitetsuppföljning på rådande rutin som jämförelse.**

**Kunskapsvinster och betydelse:**

Med hjälp av interventionerna A och B (telefonsamtal, respektive HPV hemtest ) förväntar vi oss att öka andelen kvinnor som lämnar cellprov/HPV-test jämfört med kontrollgruppen C ( årlig påminnelse) och på det sättet få möjlighet att upptäcka och behandla cellförändringar i tid innan de eventuellt utvecklas till cervixcancer. De cellförändringar som upptäcks kommer att handläggas enligt klinikens rutiner och vårdprogram.

Vi får också veta om någon av våra interventioner leder till ökat deltagande i uteblivargruppen och det kan i så fall i förlängningen implementeras i den kliniska vardagen.

Projektet kan också vara vägledande för andra vårdgivare i hur man ska uppmärksamma uteblivarna.

Vi planerar att göra en interims analys efter att hälften av kvinnorna inkluderats i studien. Om den ena armen visar sig överlägsen någon av de andra kommer studien att avbrytas.

**Utförare av projektet( se ekonomisk kalkyl)**

Vårdadministratör ca 60%

Virologen utför stor del av arbetet med HPV hemtest armen

Barnmorska för samtal i telefon

Projektledare

**Referenslista:**

1.The Global Burden of Cancer 2013. Global Burden of Disease Cancer Collaboration, Fitzmaurice C et.al. JAMA Oncol. 2015 Jul;1(4):505-27. doi: 10.1001/jamaoncol.2015.0735.

2.Socialstyrelsen. Cancer incidensen i Sverige 2010.

3. Human papillomavirus and cervical cancer. Schiffman M, Castle PE, Jeronimo J, Rodriguez AC, Wacholder S. Lancet. 2007 Sep 8;370(9590):890-907.

4. Broberg. Non-attendees need attention. Avhandling.Göteborgs universitet 2014.

5. Predictors of non-participation in cervical screening in Denmark. Kristensson JH, Sander BB, von Euler-Chelpin M, Lynge E.Cancer Epidemiol. 2014 Apr;38(2):174-80. doi: 10.1016/j.canep.2013.12.007. Epub 2014 Jan 18.

6. Cervical screening participation and risk among Swedish-born and immigrant women in Sweden. Azerkan F, Sparén P, Sandin S, Tillgren P, Faxelid E, Zendehdel K. Int J Cancer. 2012 Feb 15;130(4):937-47. doi: 10.1002/ijc.26084. Epub 2011 Jun 16.
