# Supplementary material for: Participation in interventions and recommended follow-up for non-attendees in cervical cancer screening -taking the women’s own preferred test method into account—A Swedish randomised controlled trial
Source: PLoS One. 2020 Jul 2;15(7):e0235202. doi: 10.1371/journal.pone.0235202 (PMC7332065; doi:10.1371/journal.pone.0235202)
Supplement: S2 Data — (DOCX) [file pone.0235202.s003.docx]

**Komplettering till ansökan om**

**Riktade insatser till uteblivarna i cervixcancerscreeningprogrammet.**

**Dnr 2015/480-31**

**Till Prof Charlotta Dabrosin Vetenskaplig sekreterare i regionala etikprövningsnämnden i Linköping**

Forskningsplanen är kompletterad med ytterligare information i fet stil. Något forskningsprotokoll/CRF för varje patient är inte aktuellt i denna studie.

Forskningsprojektet omfattar i huvudsak tre patientinformationer (2 inbjudningar och ett påminnelsebrev) vilka har kompletterats med föreslagen information och bifogas detta brev. De övriga breven ingår i den fortsatta handläggningen om patienten accepterar deltagande i studien och kan enligt er utgå från ansökan.

Samtliga uteblivare från cervixcancer screeningprogrammet är redan identifierade då Kvinnokliniken har fått i uppdrag av Region Östergötland att uppmärksamma dessa och öka provtagningen genom olika åtgärder vilka är beskrivna i forskningsplanen. De kvinnor som randomineras till årlig påminnelse, vilket är rådande rutin på Kvinnoklinikerna i Östergötland, kommer ej informeras om att de ingår i en kontrollgrupp. Uppgifter kring om de lämnat cellprov och resultatet av detsamma kommer inhämtas från nationella kvalitetsregistret efter godkännande från er, data kommer dock vara avidentifierade och redovisas på gruppnivå. Detta kan också ses som en kvalitetskontroll på rådande rutin.

**Caroline Lilliecreutz**

**Mödrahäslovårdsöverläkare Kvinnokliniken US**

**Anna-Clara Spetz-Holm**

**Universitetsöverläkare Kvinnokliniken US**
