# Supplementary material for: Participation in interventions and recommended follow-up for non-attendees in cervical cancer screening -taking the women’s own preferred test method into account—A Swedish randomised controlled trial
Source: PLoS One. 2020 Jul 2;15(7):e0235202. doi: 10.1371/journal.pone.0235202 (PMC7332065; doi:10.1371/journal.pone.0235202)
Supplement: S3 Data — (DOCX) [file pone.0235202.s004.docx]

**Additional information to the first application**

**Dnr 2015/480-31**

**To Professor Charlotta Dabrosin Head of the Regional Ethical Board in Linköping**

Additional Information is added in bold type in the research plan. We are not using a CRF/protocol for each study participant.

The project have mainly three information letters to the study participant (2 invitation letters and one remainder letter) these has been changed according to your decision. The rest of the letters are used in the clinical routine if the person accept to participate and do not have o be part of this application according to our opinion.

All non-attendee of the cervical cancer screening program are already identified since the Women’s clinic had an assignment from the head of the region to increase the participation rate with intervention described in the research plan. The women that are randomized to the control group with a yearly invitation (according to current routine) will not be addressed in the study. Data on Pap smear will be extracted from the national registry of cervical cancer they will be unidentified and results will presented on group level.

**Caroline Lilliecreutz**

**Mödrahälsovårdsöverläkare Kvinnokliniken US**

**Anna-Clara Spetz-Holm**

**Universitetsöverläkare Kvinnokliniken US**
