# Supplementary material for: Participation in interventions and recommended follow-up for non-attendees in cervical cancer screening -taking the women’s own preferred test method into account—A Swedish randomised controlled trial
Source: PLoS One. 2020 Jul 2;15(7):e0235202. doi: 10.1371/journal.pone.0235202 (PMC7332065; doi:10.1371/journal.pone.0235202)
Supplement: S4 Data — (DOCX) [file pone.0235202.s005.docx]

**Project plan**

**Interventions to non –attendees in the cervical cancer screening program**

Cervical cancer is the fourth most common cancer among women with 485 000 new cases world wide 2013 and the cause of 236 000 deaths (1). Since the introduction of organized cervical cancer screening programs in the 1960´s the incidence in Sweden has decreased. About 450 women/year are diagnosed with cervical cancer in Sweden and during 40 years with screening program the incidence has decreased with 67 %(2).

A high participation rate in the cervical cancer screening program is a determine factor to prevent the occurrence of high-grade intraepithelial cervical lesion and provides a nearly 90% protection for developing the cancer (3). The prerequisite for developing dysplasia and cancer in the cervix is a persistent infection of human papilloma virus (HPV). Studies shows that approximately 80% of those who die from cervical cancer and two thirds of those who develop cervical cancer has not been compliant to the cervical cancer screening program. The occurrence of high grade dysplasia is 4 times higher in the group that do not attend the recommended screening intervals compared to those who do (4).

There are different reasons for women to decline participation when invited for a Pap smear; anxiety, the feeling of having no problems, uncomfortable in the situation or fear of having cancer. The accessibility to the Pap smear clinic is of importance. The participation rate in the cervical cancer screening program is lower amongst single, women with lower education and belonging to a low socioeconomic group (5). Immigrants to Sweden also participate less in the program (6).

The definition of a non attendee found in other studies are women who has not participated with a pap smear for more than 6 years in the age group 30-49 years and more than 8 years in the age group of 50-65 years. These women is a risk group for receiving cervical cancer and it is of great importance with intervention in order to increase the attendees to the cervical cancer screening program (4).

The Swedish national cervical cancer screening registry contains historical data of Pap smear and treatment for all women identified by their unique personal number. Region Östergötland has since 2015 connection to the online part of the registry to be able to identify which women that does not attend as recommended. Data from 2012 indicates that the number of non-attended are about 12 000 women in our region.

**The aims with the project are:**

- to increase the participation among the non attendees and in that way be able to offer a high risk group of women testing and treatment.

-to examine what intervention that contributes to the highest participation rate along with a sample

-to examine the presence of dysplasia in the different interventions groups (see further down)

-to examine attendance to recommended follow up

- to examine the reasons for not having attended historical screening

**Method:**

We aim to randomize approximately 12000 women defined as non-attendees in the region of Östergötland to the following 3 groups:

1. HPV self-test: Number: 4000. The women receive a letter with studyinformation and a HPV self-test delivered to their home address. If the test has not been returned within one month a reminder letter is sent. Women with positive HPV receive a letter with information about the result and a date and time for a visit with a gynaecologist to for Pap smear and colposcopy (examination of the cervix using a microscope). Women with negative HPV receive a letter with the result and a recommendation to attend the next Pap smear invitation (yearly invitation as long as no pap smear is registered).
2. Telephone group : Number: 4000 If a telephone number can be identified from different sources (cosmic/obstetrix/internet) the women in this group receives an information letter by post that a midwife will call them within one month. During the phone call the midwife has guidelines as support. The women can choose between a visit with a midwife for a Pap smear or receiving an HPV self-test. The women that chose an HPV self-test will be handled according to A. The women that prefer a visit for a Pap smear test recieves the time and place directly from the midwife. The women that decline both offers will be recommended to participate in the future (yearly invitation). If no telephone number can be find the women will only have a yearly invitation (C).
3. Control group: Number: 4000. **This group will ha a yearly invitation for Pap smear according to the existing routine at the women clinic. This invitation will also be distributed to group A and B. No information letter will be send to this group. We wish to be able to analyse the number of Pap smears and the results from the Pap smears in this group during two years after the start of the study for comparison.**

**Gain of knowledge and importance:**

We expect that interventions A and B (Telephone Group and HPV self-test Group) increases the number of women that participate compared to group C. We can then be able to diagnose and treat dysplasia of the cervix before they progress to cervical cancer. The women with dysplasia will be treated according the medical routine.

If one of the interventions is better than the rest that could be implemented as a clinical routine and also spread among other caregivers.

Analyses during the study will be carried out for safety reasons.

**Project group**

Administrator (60% of full time)

Virology for HPV self- test analyses

Midwife for Telephone group

Head of the project

**Reference:**

1.The Global Burden of Cancer 2013. Global Burden of Disease Cancer Collaboration, Fitzmaurice C et.al. JAMA Oncol. 2015 Jul;1(4):505-27. doi: 10.1001/jamaoncol.2015.0735.

2.Socialstyrelsen. Cancer incidensen i Sverige 2010.

3. Human papillomavirus and cervical cancer. Schiffman M, Castle PE, Jeronimo J, Rodriguez AC, Wacholder S. Lancet. 2007 Sep 8;370(9590):890-907.

4. Broberg. Non-attendees need attention. Avhandling.Göteborgs universitet 2014.

5. Predictors of non-participation in cervical screening in Denmark. Kristensson JH, Sander BB, von Euler-Chelpin M, Lynge E.Cancer Epidemiol. 2014 Apr;38(2):174-80. doi: 10.1016/j.canep.2013.12.007. Epub 2014 Jan 18.

6. Cervical screening participation and risk among Swedish-born and immigrant women in Sweden. Azerkan F, Sparén P, Sandin S, Tillgren P, Faxelid E, Zendehdel K. Int J Cancer. 2012 Feb 15;130(4):937-47. doi: 10.1002/ijc.26084. Epub 2011 Jun 16.
